# Evaluation of the in vitro effects of concentrations of antibiotics on three Enterobacteriaceae isolates

**DOI:** 10.1007/s11274-023-03877-w

**Published:** 2024-01-19

**Authors:** Eda Delik, Berfin Eroğlu, Burcu Emine Tefon-Öztürk

**Affiliations:** Biology Department, Faculty of Science, Akdeniz University, 07070 Antalya, Turkey

**Keywords:** Antibiotics, *Klebsiella quasivariicola*, *Pantoea agglomerans*, *Raoultella ornithinolytica*, Sub-MIC, Virulence factors

## Abstract

Due to the misuse and overuse of antibiotics, bacteria are now exposed to sub-minimum inhibitory concentrations (sub-MICs) of antibiotics in various environments. In recent years, exposure of bacteria to sub-MICs of antibiotics has led to the widespread emergence of antibiotic-resistant bacteria. In this study, three bacterial species from the Enterobacteriaceae family (*Raoultella ornithinolytica, Pantoea agglomerans* and *Klebsiella quasivariicola*) were isolated from water. The antibiotic susceptibility of these bacteria to 16 antibiotics was then investigated. The effects of sub-MICs of four selected antibiotics (kanamycin, chloramphenicol, meropenem, and ciprofloxacin) on the growth, biofilm formation, surface polysaccharide production, siderophore production, morphology, and expression of the translational/transcriptional regulatory transformer gene *rfaH* of these bacteria were analysed. The MICs of kanamycin, chloramphenicol, meropenem, and ciprofloxacin were determined to be 1, 2, 0.03 and 0.03 µg/mL for *R. ornithinolytica*; 0.6, 6, 0.03 and 0.05 µg/mL for *P. agglomerans*; and 2, 5, 0.04 and 0.2 µg/mL for *K. quasivariicola*. The growth kinetics and biofilm formation ability decreased for all three isolates at sub-MICs. The surface polysaccharides of *R. ornithinolytica* and *P. agglomerans* increased at sub-MICs. There was no significant change in the siderophore activities of the bacterial isolates, with the exception of MIC/2 meropenem in *R. ornithinolytica* and MIC/2 kanamycin in *K. quasivariicola*. It was observed that the sub-MICs of meropenem and ciprofloxacin caused significant changes in bacterial morphology. In addition, the expression of *rfaH* in *R. ornithinolytica* and *K. quasivariicola* increased with the sub-MICs of the selected antibiotics.

## Introduction

Members of the Enterobacteriaceae are found in many places in nature, including soil, water, termites, nematodes, insects, plants and vertebrates. The clinical importance of this family is great and includes several potentially pathogenic species (e.g. *Salmonella*, *Shigella* and *Yersinia*) as well as opportunistic pathogens (e.g. *Klebsiella pneumoniae, Raoultella ornithinolytica* and *Pantoea agglomerans*). Other bacterial groups identified to date do not have as great an impact on medicine, public health and veterinary medicine as the Enterobacteriaceae family. This family is not only associated with a variety of clinical syndromes, but is also an important causative agent of foodborne enteritis and zoonotic infections (Janda and Abbott 2021).

The genus *Klebsiella*, an important genus in the Enterobacteriaceae family, is a non-spore-forming, encapsulated, Gram-negative, aerobic, and facultative anaerobic bacteria. This genus includes several species that cause nosocomial infections and community-acquired infections. Among the members of the genus, *K. pneumoniae* is the predominant cause of human infections. This species is closely related to *K. quasipneumoniae* subsp. *quasipneumoniae*, *K. africaensis*, *K. quasipneumoniae* subsp. *similipneumoniae*, *K. variicola*, *K. variicola* subsp. *tropicalensis* and *K. quasivariicola*, which together form the *K. pneumoniae* species complex (Håkonsholm et al. 2020). *K. quasivariicola* is one of the recently described species and was obtained from a wound culture (Long et al. 2017). Information on this new species is quite limited. This species possesses broad-spectrum β-lactamase enzyme genes that are important for its virulence and can therefore cause severe infections in humans (Long et al. 2017).

The genus *Raoultella* is a non-spore-forming, Gram-negative, encapsulated, aerobic and facultative anaerobic bacteria in the Enterobacteriaceae. It currently consists of four species, *R. ornithinolytica, R. electrica, R. terrigena,* and *R. planticola*. Previously, these species were categorized under the genus *Klebsiella*. However, the current taxonomy was later created established based on a comparative analysis of phylogenetic analyses, leading the creation of the new genus *Raoultella* (Drancourt et al. 2001). Among the members of the genus, *R. ornithinolytica* is a histamine-producing bacterium; therefore, it has become a foodborne pathogen of major importance and has been associated with histamine poisoning in humans (Hajjar et al. 2020; Abd El-Ghany 2021). Infections have also been reported to cause pneumonia, urinary tract infections and biliary tract disease in humans (Van Cleve et al. 2018; Abd El-Ghany 2021).

The genus *Pantoea* is a Gram-negative, non-encapsulated, non-spore-forming, aerobic and facultative anaerobic bacteria of the Enterobacteriaceae family. Among the members of the genus, *P. agglomerans* is the most frequently isolated species associated with human infections. This bacterium is generally considered opportunistic, of low virulence and low toxicity. However, it has been associated with important human diseases such as meningitis, pneumonia, wound infections, urinary tract infections, lung and brain abscesses, osteomyelitis and septicaemia (Mardaneh and Dallal 2013). There is limited information on possible determinants of the virulence of this bacterium, which remain largely unexplored (Guevarra et al. 2021). This bacterium produces a biologically potent endotoxin (lipopolysaccharide) (Dutkiewicz et al. 2016). In addition, some genes have been linked to the increased virulence and pathogenesis of these bacteria. The proteins encoded by these genes inhibit the host's immune response and manipulate its cellular functions (Abdulamir et al. 2020).

Antibiotic therapy is available for Enterobacteriaceae infections, and a rapid increase in antibiotic resistance has been reported among members of this family (Seng et al. 2016; Church and McKillip 2021). A study conducted in 2021 found that urine samples from a clinical setting contained predominantly bacterial isolates belonging to Enterobacteriaceae, and these isolates exhibited multidrug resistance (Bayode et al. 2021). Antibiotic resistance is an important universal health challenge, causing significant morbidity or mortality (Darby et al. 2023). Therefore, it is crucial to discover alternative antimicrobial agents, understand the mechanisms of antimicrobial resistance, and take measures to contain the spread of resistant microorganisms (Olusola-Makinde and Bayode 2021; Bayode et al. 2021). Antibiotic doses encountered by bacteria during treatment are high, but antibiotic concentrations released from anthropogenic sources are generally below minimum inhibitory concentrations (MICs) (Stanton et al. 2020). Antibiotic-sensitive bacteria die or stop growing at antibiotic concentrations above the MIC. In contrast, bacteria can still grow at antibiotic doses below the MIC. This could potentially create a different evolutionary trajectory with the accumulation of resistance mutations in bacteria (Wistrand-Yuen et al. 2018). There are many studies on the effects of sub-MICs on bacterial cells and it has been reported that these doses can cause changes in bacterial virulence, gene expression and gene transfer (Liu et al. 2019; Navidifar et al. 2019; Shenkutie et al. 2022; Berryhill et al. 2023; Delik et al. 2023).

In this study, three Enterobacteriaceae species (*R. ornithinolytica*, *P. agglomerans*, *K. quasivariicola*) were identified from a freshwater sample. The antibiotic susceptibility of these isolates was investigated, and the MIC values were determined. In addition, the effects of sub-MICs (1/2 and 1/4) of 4 selected antibiotics (kanamycin, meropenem, chloramphenicol, ciprofloxacin) on the growth rate, biofilm formation, surface polysaccharide production, siderophore production, bacterial morphology, and expression of the translational/transcriptional regulatory transformer gene *rfaH* of these isolates were investigated.

## Materials and methods

### Study area, sampling and bacteria isolation

The isolates used in this study were isolated from a freshwater source that is important for the province of Antalya (Turkiye) due to frequent human activities (e.g., swimming and fishing) and its use for the irrigation of agricultural land (geographical coordinates 36°51′12.5″N 30°37′39.1″E). Water samples were collected in sterile falcon tubes. Then the samples were immediately transported to the laboratory in a cool box.

50 mL water samples were serially diluted (1:10) with 0.85% NaCl. Then 200 µL of the suspension from each dilution was transferred to a nutrient agar (NA) by using spread plate technique and incubated at 30˚C for 96 h. Subsequently, the selected colonies were transferred to NA, and subcultures and − 80˚C stocks were prepared for further studies.

### Identification of the isolates by 16S rRNA sequence analysis

Genomic DNA was isolated using a commercial extraction kit (Zymo Research, USA) for molecular identification of some selected isolates. Universal primers were used for PCR amplification of the 16S rRNA region (8F and 1492R) and the PCR reactions were conducted following previously established protocol (Eden et al. 1991). Samples were bidirectionally sequenced using the BigDye Terminator Cycle Sequencing Kit on the ABI 3730XL Genetic Analyser. The results were aligned and isolates identified using the Basic Local Alignment Search Tool (BLASTn) programme (Benson et al. 2000) of the National Centre for Biotechnology Information (NCBI) database.

### Antibiotic susceptibility and MIC testing

Overnight cultures of isolates were adjusted to 0.5 McFarland turbidity standard in NaCl (0.85%), and the antibiotic sensitivity screening conducted as described by the Clinical and Laboratory Standards Institute (CLSI 2021). Sterile swabs were used to inoculate the bacteria onto Mueller Hinton Agar (MHA). The antibiotic discs (kanamycin (30 µg), imipenem (10 µg), chloramphenicol (30 µg), meropenem (10 µg), gentamicin (10 µg), meropenem (10 µg), tetracycline (30 µg), ceftriaxone (30 µg), ciprofloxacin (5 µg), ceftazidime (30 µg), cefuroxime (30 µg), cefazolin (30 µg), ampicillin (10 µg), erythromycin (15 µg), azithromycin (15 µg), clarithromycin (15 µg)) and trimethoprim-sulfamethoxazole (TMP-SMX) (1.25/23.75 µg) were added to the MHA inoculated with bacteria. The petri dishes were placed in an incubator at 37 °C for 24 h, and the inhibition zone diameter was subsequently measured. Sensitive, intermediate and resistant isolates were identified according to CLSI standards.

Determination of MIC of the antibiotics on the bacterial isolates was done using the broth dilution method as explained by CLSI (2021) with different concentrations of the antibiotic. Overnight cultures of the isolates were adjusted to the 0.5 McFarland turbidity standard. Following this, antibiotic stock solutions were serially diluted, and the bacterial cultures were cultivated using these antibiotic dilutions. Antibiotic-free bacterial cultures were used as positive control and NB medium as negative control and incubated at 37 °C and 150 rpm for 24 h. The minimum concentration of antibiotic that inhibited bacterial growth was designated as MIC.

### Bacterial growth

Growth curves were generated to determine the effect of sub-MICs of antibiotics on the growth rate of the isolates. The concentrations of the fresh isolates were adjusted to 0.02 (at OD_600_) in NB. Bacterial cultures containing antibiotics in 1/2 and 1/4 sub-MIC doses were established. Antibiotic-free bacterial cultures were used as positive controls and incubated at 37 °C and 150 rpm for 24 h. OD_600_ values were measured and recorded every 2 h for 24 h.

### Bacterial biofilm formation

The crystal violet method was used with minor modifications to assess the biofilm formation of bacteria. (Tang et al. 2020). The concentrations of fresh isolates were adjusted to 0.05 (at OD_600_) in NB and antibiotic-containing bacterial cultures were grown at sub-MICs (1/2 and 1/4) of the MICs determined and 200 µL were inoculated into 96-well plates. The antibiotic-free bacterial culture served as a positive control and the sterile NB was used as a negative control. The plates incubated at 37 °C for 24 h. Later, the plates were washed with distilled water and then air-dried. Then 200 µL of crystal violet (0.1%) was added to each well and the plates were incubated for 30 min. The plates were washed again using sterile water and air dried. The adhering bacteria were quantified by adding ethanol (95%) and measuring the dissolved crystal violet at an optical density of 590 nm.

### Quantification of surface polysaccharides

Surface polysaccharide production was determined using a phenol–sulphuric acid method as previously described (Brimacombe and Beatty 2013). Briefly, 1 mL of the overnight cultures was harvested and centrifuged at 14,500 rpm for 10 min. The cell pellets were washed five times with 1 mL of NaCl (50 mM). After the last wash, the cell pellets were dissolved in 1 mL of EDTA (50 mM) and incubated at 37 °C for 2 h. After incubation, the cells were pelleted by centrifugation at 14,500 rpm for 10 min and the supernatant was carefully removed. 400 µL of the supernatant and for blanking, 400 µL of EDTA (50 mM) were transferred to a clean glass tube. 400 µL of phenol (5%) and 2 mL of sulphuric acid (95%) were carefully added to each tube. The reaction was mixed gently for 10 min at room temperature. The OD_490_ of all reactions was measured with a spectrophotometer and normalised to the standard curve to calculate the carbohydrate concentration.

### Siderophore production

To assess siderophore production, the method described by Lovato et al. (2019) was used, with some modifications. Fresh cultures of isolates grown in NB medium were adjusted to 0.02 (at OD_600_), inoculated in NB medium as control or NB, supplemented with an antibiotic corresponding to sub-MIC of 1/2 or 1/4 µg/mL, and incubated at 37 °C for 24 h, shaking at 150 rpm. Then 10 µL of each bacterial culture was seeded on CAS agar and incubated for 24 h at 37 °C. After incubation, the diameters of the yellow halos were measured.

### Bacterial morphological observations

The effect of sub-MICs of antibiotics on morphology was evaluated using light microscopy. Concentrations of fresh isolates were adjusted to 0.02 (at OD_600_) at NB. Antibiotic-containing bacterial cultures were prepared at 1/2 and 1/4 sub-MICs and incubated for 24 h at 37 °C and 150 rpm. The bacterial cultures obtained after passage under sub-MICs of antibiotics were subjected to Gram staining to observe any noticeable changes in cell morphology. The antibiotic-free bacterial culture was used as a positive control.

### RNA isolation and gene expression

To analyse the effect of sub-MICs on the expression of virulence gene (*rfaH*), total RNA of bacteria was extracted with the Zymo Research Total RNA isolation kit. The cDNA conversion of the isolated RNA was performed using a cDNA synthesis kit from Jena Bioscience according to the manufacturer’s instructions. Real-time PCR analyses were performed using the QuantiNova SYBR Green PCR Kit and run in the RotorGene Q 5plex HRM instrument (Qiagen, Hilden, Germany). All primers used for the target genes and the housekeeping gene (*rho*) are listed in Table 1. Each reaction consisted of 1 µL of each primer (100 µM), 10 µL SYBR Green Master Mix, 1 µL cDNA, and 7 µL sterile water. The reaction initiated with a denaturation step at 95 °C for 2 min, followed by 40 amplification cycles with denaturation at 95 °C for 5 s, annealing and extension at 60 °C for 10 s. The expression levels of antibiotic-treated cultures were compared with those of antibiotic-free cultures, and the data were analysed using the 2^−ΔΔCT^ method.

**Table 1:** Synthesized oligonucleotide primer sequences

Genes	Primers	Amplicon size (bp)	References
*rfaH* (*R. ornithinolytica* and *K. quasivariicola*)	Forward: 5′- TCAACGAGCCCAGGAACATC-3′ Reverse: 5′- CCAAAAGCGCACGAAGTTGT -3’	205	This study
*rfaH* (*P. agglomerans*)	Forward: 5′- ATCGAGTTTGATCCGGAAGCC-3’ Reverse: 5′- CTTCAAACGTCCCGTCGGTG -3′	205	This study
*rho*	Forward: 5′-AACTACGACAAGCCGGAAAAC-3′ Reverse: 5′- AGAACCGTTACCACGCTCCA-3′	102	Gomes et al. 2018

### Statistical assessments

The experiments were carried out in triplicate in three separate experiments. The data obtained are expressed as mean ± standard deviation. Data were analysed using IBM SPSS 22 software (SPSS, USA) and statistical analysis was performed using one-way analysis of variance (ANOVA) and Tukey’s test for multiple comparisons, with p < 0.05 considered statistically significant.

## Results

### Sequence analysis

Three different isolates from the freshwater sampling area, belonging to the Enterobacteriaceae family were identified as: *R. ornithinolytica* (GeneBank ID: NR_044799.1), *P. agglomerans* (GeneBank ID: NR_041978.1) and *K. quasivariicola* (GeneBank ID: NR_181901.1).

### Antibiotic susceptibility testing

*R. ornithinolytica* and *K. quasivariicola* were found to be resistant to 4 (ampicillin, azithromycin, clarithromycin, and erythromycin) of the 16 antibiotics, and in addition *R. ornithinolytica* showed intermediate resistance to 2 (gentamicin and kanamycin) antibiotics (Table 2). *P. agglomerans* was sensitive to all antibiotics used in this study and was only intermediate resistant to 2 antibiotics (ampicillin and kanamycin).

**Table 2:** Antibiotic sensitivity of bacterial isolates

	*R. ornithinolytica*	*P. agglomerans*	*K. quasivariicola*
Ampicillin	R	I	R
Azithromycin	R	S	R
Cefazolin	S	S	S
Ceftazidime	S	S	S
Ceftriaxone	S	S	S
Cefuroxime	S	S	S
Chloramphenicol	S	S	S
Ciprofloxacin	S	S	S
Clarithromycin	R	S	R
Erythromycin	R	S	R
Gentamicin	I	S	S
Imipenem	S	S	S
Kanamycin	I	I	S
Meropenem	S	S	S
Tetracycline	S	S	S
TMP-SMX	S	S	S

*S sensitivity, I intermediate, R resistant

### MIC tests

The results of the disc diffusion test were used to determine the MIC values for the antibiotics to which the isolates were sensitive or intermediate resistant (Table 3). The highest MIC was observed against cefuroxime in *R. ornithinolytica* (15 µg/mL), against clarithromycin in *P. agglomerans* (7 µg/mL) and against cefazolin in *K. quasivariicola* (32 µg/mL). Meropenem was the antibiotic with the lowest MIC for all three isolates (0.03 µg/mL for *R. ornithinolytica* and *P. agglomerans*, 0.04 µg/mL for *K. quasivariicola*). In addition to meropenem, ciprofloxacin was also the antibiotic with the lowest MIC for *R. ornithinolytica*.

**Table 3:** MIC values of the antibiotics (µg/mL)

	*R. ornithinolytica*	*P. agglomerans*	*K. quasivariicola*
Ampicillin	–	0.5	–
Azithromycin	–	4	–
Cefazolin	2	2	32
Ceftazidime	1	0.2	0.6
Ceftriaxone	1	0.3	1
Cefuroxime	15	4	25
Chloramphenicol	2	6	5
Ciprofloxacin	0.03	0.05	0.2
Clarithromycin	–	7	–
Erythromycin	–	0.5	–
Gentamicin	0.8	0.5	0.6
Imipenem	0.06	0.06	0.07
Kanamycin	1	0.6	2
Meropenem	0.03	0.03	0.04
Tetracycline	0.8	2	2
TMP-SMX	0.19	0.19	1.5

To continue the study, we selected 4 antibiotics to which the isolates were sensitive to determine the effects of the sub-MICs of the antibiotics on the isolates. Accordingly, ciprofloxacin was selected from the nucleic acid synthesis inhibitor group, meropenem from the cell wall synthesis inhibitor group, kanamycin (for the 30S ribosomal subunit) and chloramphenicol (for the 50S ribosomal subunit) from the protein synthesis inhibitor group.

### Bacterial growth

**Fig. 1:**
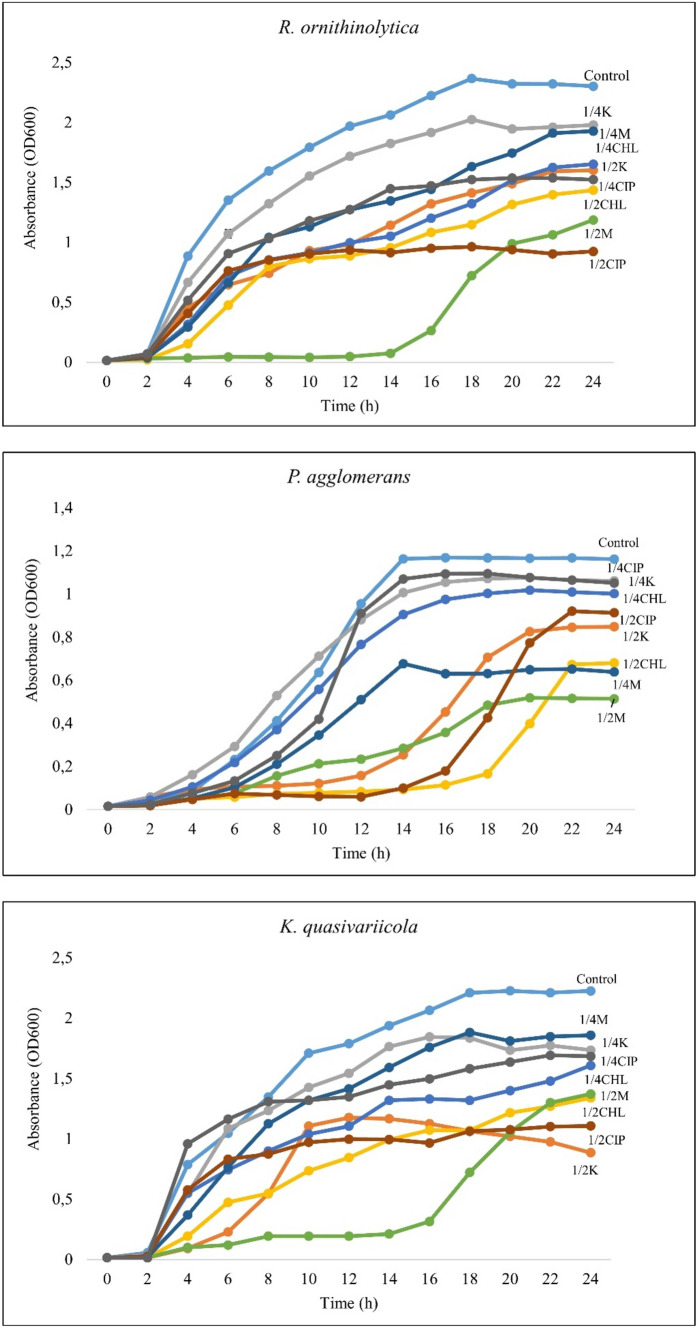
Growth curves of bacterial isolates at sub-MICs (Control: Bacterial cells grown in antibiotic-free medium, K: Kanamycin, CHL: Chloramphenicol, M: Meropenem, CIP: Ciprofloxacin)

The growth rates of the bacterial isolates and the effects of the sub-MICs on the growth rate of these bacteria are shown in Fig. 1. For *R. ornithinolytica* and *K. quasivariicola*, the highest cell concentration was reached at the 18th hour, and for these two isolates, the MIC/2 of meropenem delayed the logarithmic phase more than the other sub-MICs. Additionally, the culture of *K. quasivariicola* containing MIC/2 ciprofloxacin reached a higher cell concentration after 4 and 6 h compared to the control. For *P. agglomerans*, the highest cell concentration was reached at the 14th hour. The MIC/2 of chloramphenicol delayed the logarithmic phase more than the other sub-MICs. Furthermore, the culture of this bacterium containing MIC/4 kanamycin increased the growth rate compared to the control and became the culture with the highest cell concentration by the 10th hour.

### Bacterial biofilm formation

**Fig. 2:**
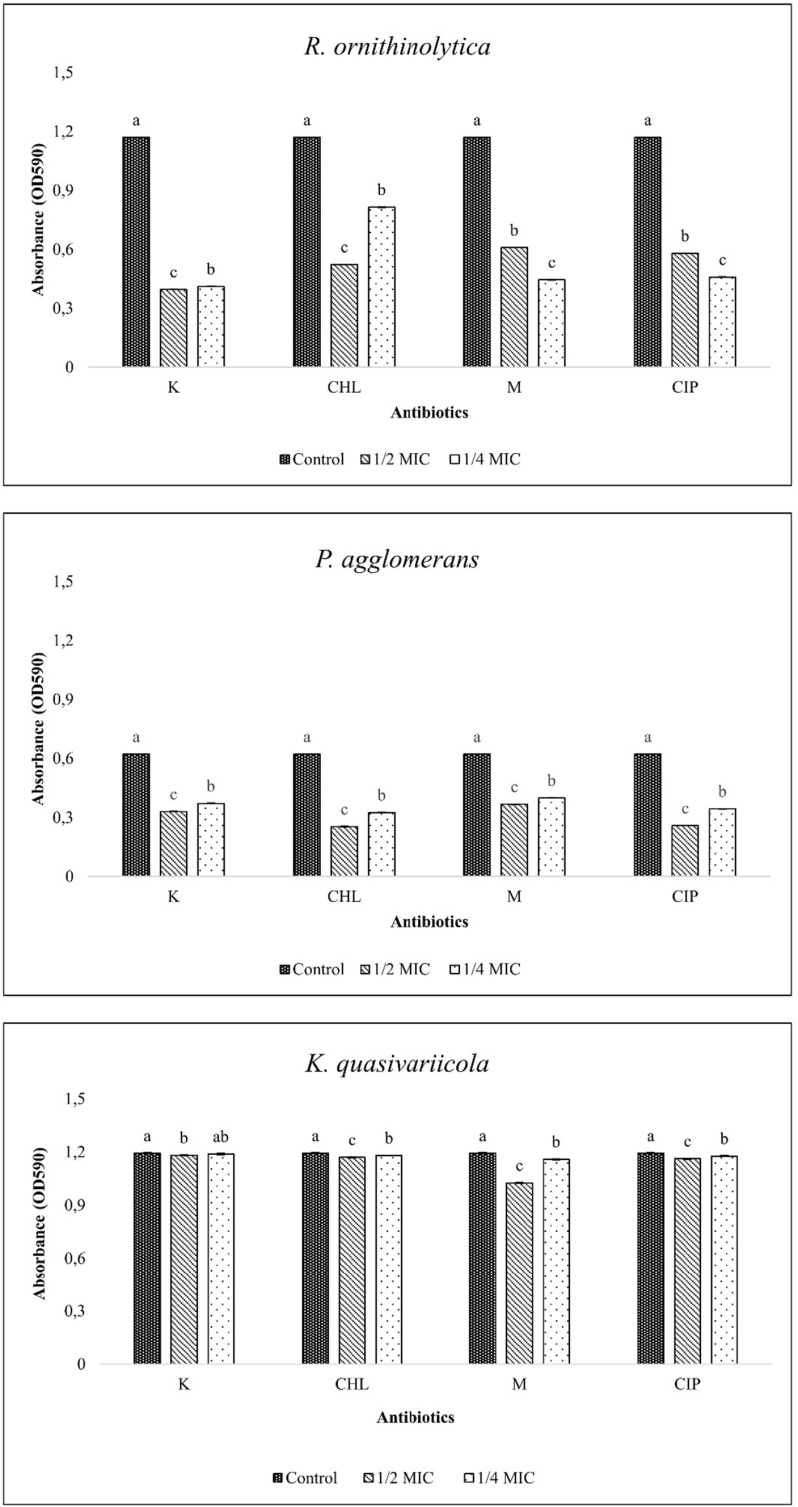
Biofilm produced by bacterial isolates on sub-MICs (Control: Bacterial cells grown in antibiotic-free medium, K: Kanamycin, CHL: Chloramphenicol, M: Meropenem, CIP: Ciprofloxacin, statistical differences are indicated by different superscript letters (p<0.05))

When the biofilm-forming capacities of the isolates were analysed, it was observed that *P. agglomerans* had notably lower biofilm production compared to the other two isolates (Fig. 2). Moreover, the sub-MICs of the antibiotics have generally significantly inhibited the biofilm production of all isolates (p < 0.05). For *P. agglomerans* and *K. quasivariicola*, the MIC/2 levels of the antibiotics were more effective than the MIC/4 values in inhibiting biofilm formation. However, this inhibition was not significant in *K. quasivariicola* with sub-MICs of kanamycin (p > 0.05). Interestingly, in contrast to kanamycin and chloramphenicol, the MIC/4 doses of meropenem and ciprofloxacin in *R. ornithinolytica* inhibited biofilm formation more effectively than the MIC/2 doses (p < 0.05).

### Surface polysaccharide extraction

**Fig. 3:**
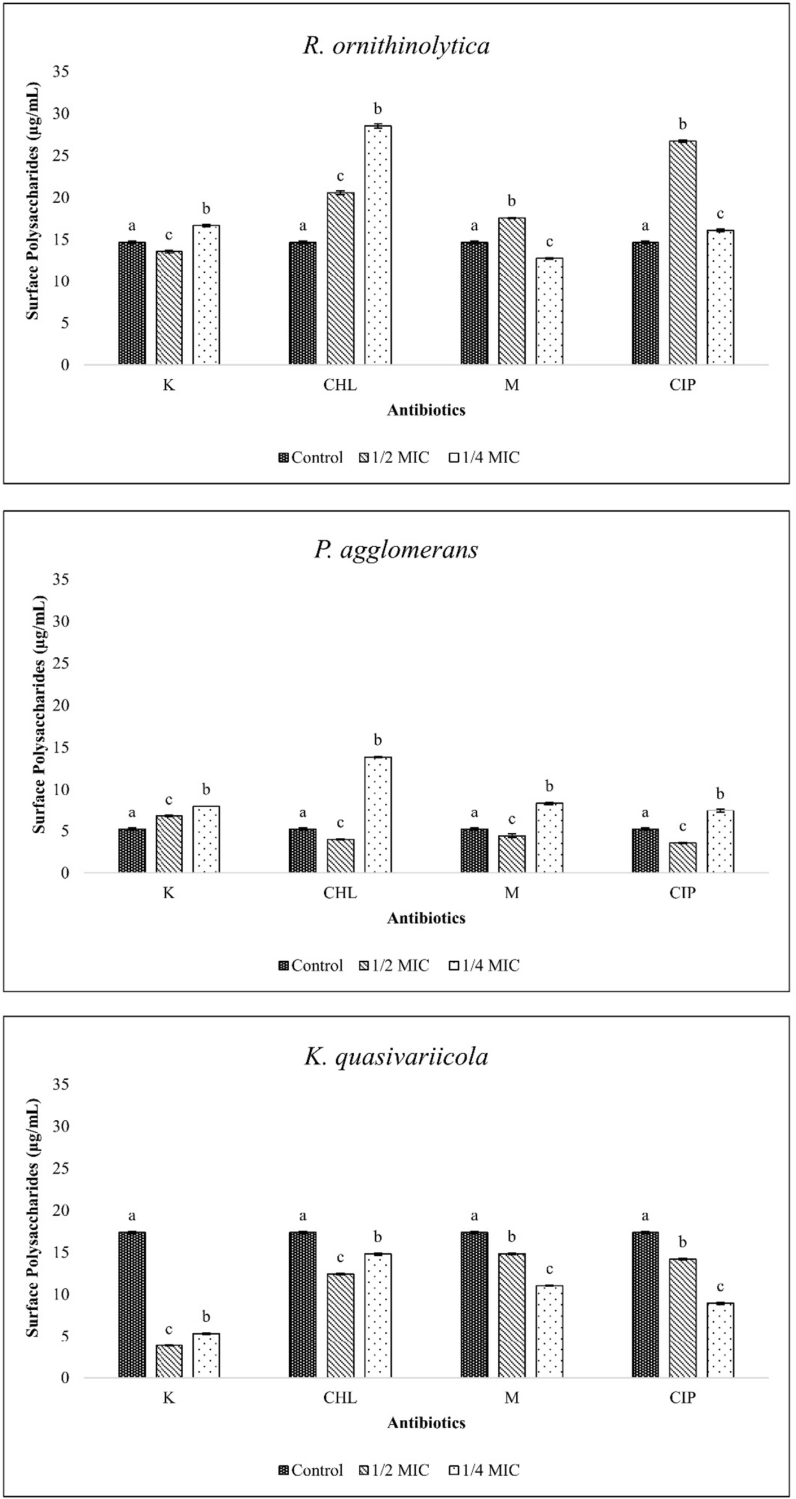
Values of surface polysaccharide extracted from bacterial isolates at sub-MICs (Control: Bacterial cells grown in antibiotic-free medium, K: Kanamycin, CHL: Chloramphenicol, M: Meropenem, CIP: Ciprofloxacin, statistical differences are indicated by different superscripts (p<0.05))

Similar to the biofilm experiment, *P. agglomerans* was able to produce fewer surface polysaccharides than the other two isolates (Fig. 3). Additionally, in this bacterium, sub-MICs of kanamycin and MIC/4 doses of other antibiotics caused a higher surface polysaccharide content compared to the control (p < 0.05). In *R. ornithinolytica*, MIC/4 kanamycin and chloramphenicol as well as MIC/2 meropenem and ciprofloxacin caused an increase in surface polysaccharides. In *K. quasivariicola*, antibiotics with sub-MICs caused a decrease in surface polysaccharides.

### Siderophore production

**Fig. 4:**
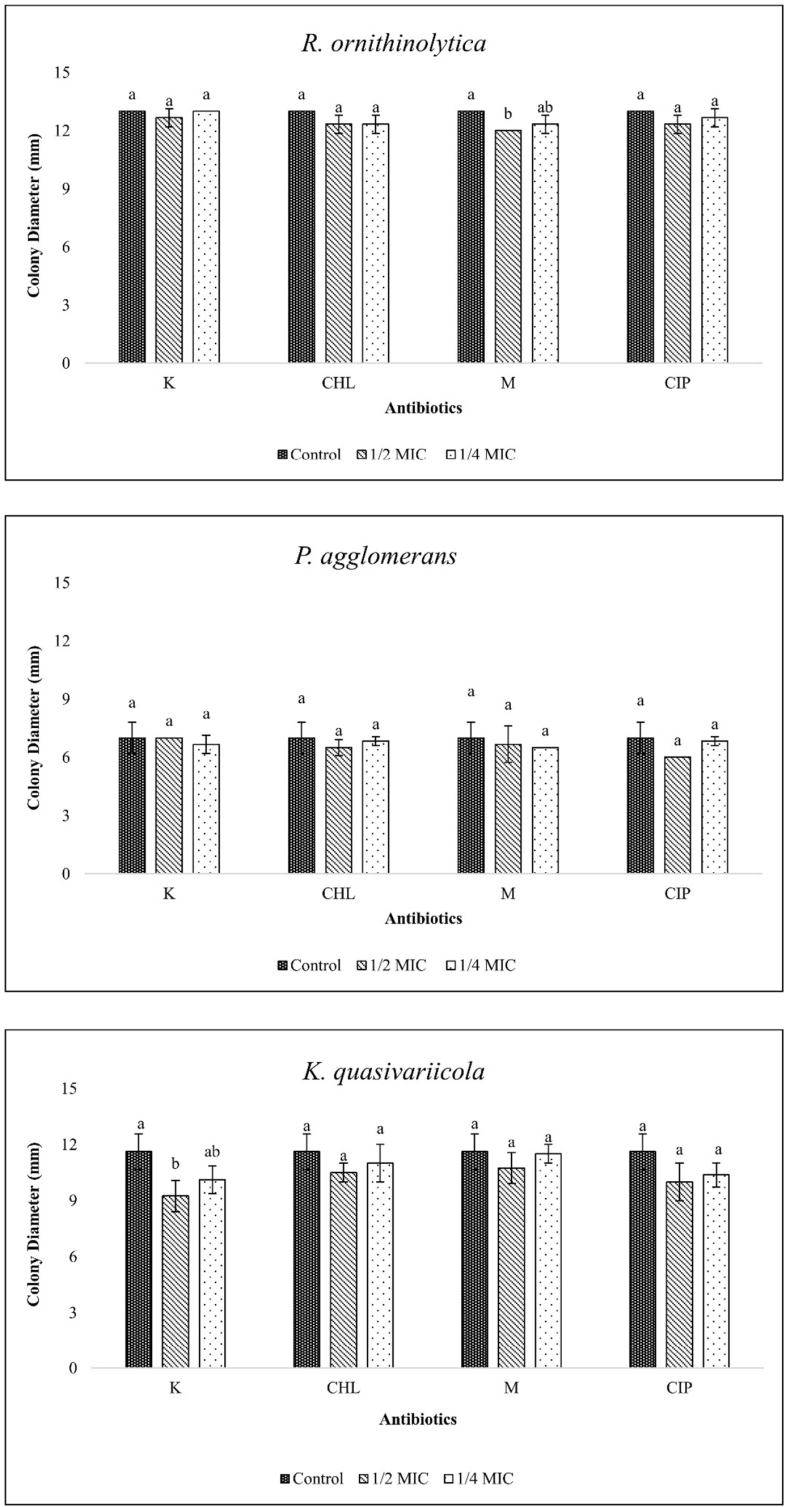
Siderophore production of bacterial isolates at sub-MICs (Control: Bacterial cells grown in antibiotic-free medium, K: Kanamycin, CHL: Chloramphenicol, M: Meropenem, CIP: Ciprofloxacin, statistical differences are indicated by different superscripts (p<0.05))

Like the biofilm and surface polysaccharide experiments, *P. agglomerans* was able to produce fewer siderophores compared to the other two isolates. Furthermore, sub-MICs had no significant effect on siderophore production of this bacterium (p > 0.05) (Fig. 4). In *R. ornithinolytica*, the MIC/2 dose of meropenem and in *K. quasivariicola*, the MIC/2 dose of kanamycin reduced siderophore production compared to the control (p < 0.05).

### Bacterial morphological observations

**Fig. 5:**
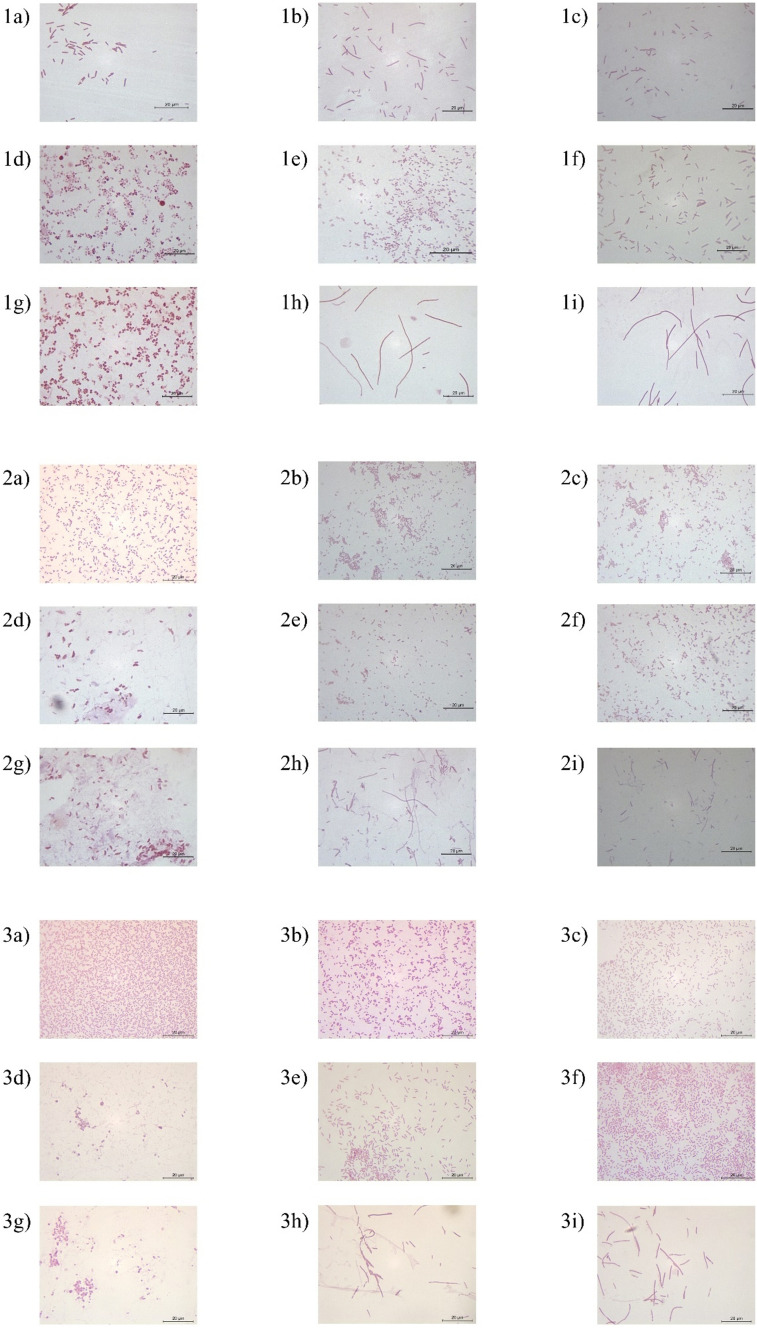
Changes in bacterial morphology in sub-MICs (1a) *R. ornithinolytica* Control: Morphology of bacteria grown in antibiotic-free media, 1b) *R. ornithinolytica* MIC/2 chloramphenicol (CHL), 1c) *R. ornithinolytica* MIC/4 CHL, 1d) *R. ornithinolytica* MIC/2 meropenem (M), 1e) *R. ornithinolytica* MIC/2 kanamycin (K), 1f) *R. ornithinolytica* MIC/4 K, 1g) *R. ornithinolytica* MIC/4 M, 1h) *R. ornithinolytica* MIC/2 ciprofloxacin (CIP), 1i) *R. ornithinolytica* MIC/4 CIP, 2a) *P. agglomerans* Control, 2b) *P. agglomerans* MIC/2 CHL, 2c) *P. agglomerans* MIC/4 CHL, 2d) *P. agglomerans* MIC/2 M, 2e) *P. agglomerans* MIC/2 K, 2f) *P. agglomerans* MIC/4 K, 2g) *P. agglomerans* MIC/4 M, 2h) *P. agglomerans* MIC/2 CIP, 2i) *P. agglomerans* MIC/4 CIP, 3a) *K. quasivariicola* Control, 3b) *K. quasivariicola* MIC/2 CHL, 3c) *K. quasivariicola* MIC/4 CHL, 3d) *K. quasivariicola* MIC/2 M, 3e) *K. quasivariicola* MIC/2 K, 3f) *K. quasivariicola* MIC/4 K, 3g) *K. quasivariicola* MIC/4 M, 3h) *K. quasivariicola* MIC/2 CIP, 3i) *K. quasivariicola* MIC/4 CIP)

The change in bacterial morphology induced by sub-MICs was observed in all three isolates. (Fig. 5). The sub-MICs of meropenem and ciprofloxacin caused the most marked morphological changes in all three bacterial isolates. In the presence of sub-MIC meropenem, the cells of *R. ornithinolytica* and *K. quasivariicola* became round, while the cells of *P. agglomerans* became comma-shaped. After treatment of the bacterial cells with sub-MIC ciprofloxacin, all bacterial isolates formed long filaments. Bacteria grown under sub-MIC conditions returned to their original morphology when cultured again in an antibiotic-free environment. Regardless of the effects of sub-MICs on cell morphology, we also observed that the cell morphology of *R. ornithinolytica* differed between agar and broth media under normal conditions. When these bacterial cells were cultured in broth, they transformed into longer bacilli that differed distinct from their morphology in agar medium. When the bacterial cells were transplanted back into the solid medium, they returned to their first form.

### Analysis of virulence gene expressions

**Fig. 6:**
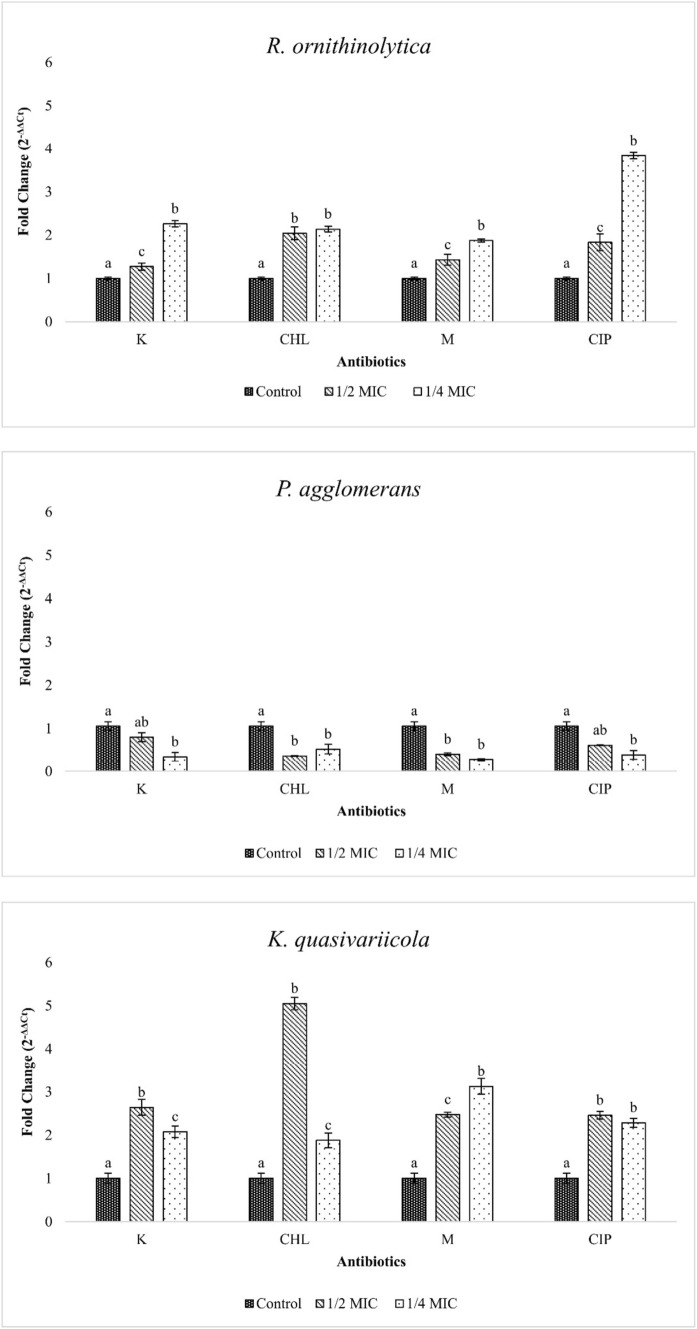
Relative expression values of *rfaH* in sub-MICs (Control: Bacterial cells grown in antibiotic-free medium, K: Kanamycin, CHL: Chloramphenicol, M: Meropenem, CIP: Ciprofloxacin, statistical differences are indicated by different superscript letters (p<0.05)

The expression level of the *rfaH* gene was affected differently in all bacterial isolates exposed to sub-MICs (Fig. 6). In both *R. ornithinolytica* and *K. quasivariicola* isolates, the expression level of the gene was significantly increased in sub-MICs compared to the control (p < 0.05). The expression of *rfaH* in *R. ornithinolytica* was significantly higher in MIC/4 than in MIC/2, except for chloramphenicol (p < 0.05). In *K. quasivariicola*, the expression of the *rfaH* gene was higher with MIC/2 of the other antibiotics, except for meropenem. Additionally, the *rfaH* gene was expressed five times higher with MIC/2 of chloramphenicol than with control (p < 0.05). In the *P. agglomerans*, the expression of *rfaH* decreased with sub-MICs of antibiotics.

## Discussion

Enterobacteriaceae is a very important family as it contains many species that threaten public health. Members of this family, which are widespread in nature, are increasingly considered to be pathogens of piscine species and the etiologic agent of a variety of plant diseases as well as various infections in humans (Janda and Abbott 2021). In this study, three species belonging to the Enterobacteriaceae family were identified from freshwater: *P. agglomerans*, which causes human disease and is commonly defined as a plant pathogen (Cruz et al. 2007), *R. ornithinolytica*, which is a major cause of foodborne illness (Hajjar et al. 2020; Abd El-Ghany 2021), and *K. quasivariicola*, a newly identified species of *Klebsiella* that is potentially pathogenic (Long et al. 2017). The clinical characteristics, antimicrobial sensitivity, and clinical consequences of infections caused by these three species are not fully understood.

In our study, we first examined the antimicrobial resistance profiles of the isolates and determined the MIC values. *R. ornithinolytica* and *K. quasivariicola* were resistant to the macrolides and ampicillin used in this study. However, no antibiotic resistance was detected in *P. agglomerans*. We then selected four antibiotics (kanamycin, meropenem, chloramphenicol, ciprofloxacin) with different mechanisms of action and studied the effects of sub-MICs of these antibiotics on the isolates. There is no study in the literature on the effects of sub-MICs on these bacterial isolates. In this study, we observed that sub-MICs significantly slowed the growth of these bacteria and delayed the logarithmic phase. Many other studies have also shown that sub-MICs have a similar effect on bacterial growth (Peng et al. 2005; Novović et al. 2018; Pitsiniaga and Sullan 2022; Sanz‐García et al. 2022; Berryhill et al. 2023; Delik et al. 2023).

In the studies we conducted, we observed that all three isolates were able to form biofilms. Biofilms are described as part of the survival mechanisms of bacteria and are considered one of the main causes of chronic infections (Vestby et al. 2020; Abebe 2020). Bacteria that form biofilms are embedded in an extracellular polymeric substance (EPS) and can be protected from harsh environmental conditions such as biocides, antibiotics, UV radiation and the host immune system (Abebe 2020). Considering the potential of low doses of antibiotics to promote biofilm formation and the development of antibiotic resistance (Shenkutie et al. 2022), we investigated the effect of sub-MICs on the ability of these bacteria to form biofilms. To deepen our understanding of biofilm formation, we also investigated the effects of sub-MICs on the formation of surface polysaccharides. Bacterial cell surface polysaccharides play an important role in biofilm formation and the expression of virulence. The polysaccharides organised on the surface are referred to as capsules with a different structure, while the released type is EPS (Xu et al. 2022). Additionally, the surface polysaccharides are considered the primary virulence factors of Gram-negative bacteria and are considered priority vaccine candidates for the pathogen (Wang et al. 2021). Previous studies have demonstrated a positive correlation between biofilm formation and EPS production in sub-MIC environments (Majtan et al. 2008; Chadha 2021). Interestingly, however, in this study we observed that sub-MICs of antibiotics can lead to a decrease in biofilm production and a concomitant increase in surface polysaccharides, especially in *R. ornithinolytica* and *P. agglomerans*. The lower biofilm formation and the lower amount of surface polysaccharides in *P. agglomerans* compared to the other two isolates could also be due to the fact that this bacterium does not form a capsule.

In addition to biofilm formation, bacteria can increase their proliferation and infectivity in the host by producing siderophores. Siderophores are components produced by bacteria to facilitate iron uptake and thereby enhance their survival and ability to multiply. Previous studies have reported that *R. ornithinolytica* can produce aerobactin (Al-Hulu et al. 2009), yersiniabactin, and enterobactin (Wang et al. 2021), and that *P. agglomerans* can produce siderophores such as enterobactin and deferoxamine E (Sulja et al. 2022). Although there is no study on siderophores produced by *K. quasivariicola*, it is known that *Klebsiella* species can produce siderophores such as enterobactin, aerobactin, and yersiniabactin (Shankar et al. 2018). In our studies, we found that all three isolates were capable of producing siderophores, and we investigated the effects of sub-MICs on bacterial siderophore production. In this study, sub-MICs generally reduced or had no effect on siderophore production. Similarly, Gupta et al. (2016), Ding et al. (2017) Lovato et al. (2019) and Durgadevi et al. (2019) reported that sub-MICs inhibited or had no effect on bacterial siderophore production.

Bacteria may show different morphologies in their original form when treated with sub-MIC antibiotics. In this study, we observed that the sub-MICs of meropenem and ciprofloxacin induce changes in bacterial cell morphology. Meropenem has a high affinity for penicillin-binding proteins (PBPs). These PBPs, which play a role in completing peptidoglycan assembly, are also involved in cell division and elongation. Binding of meropenem to these PBPs can lead to the morphology of bacterial cells to become round or oval, with a wider centre (Opstrup et al. 2023). In this study, we believe that the induction of round cell morphology by meropenem in *R. ornithinolytica* and *K. quasivariicola* and comma-shaped cell morphology in *P. agglomerans* may be due to binding to different combinations of PBPs. Ciprofloxacin, on the other hand, caused the formation of long filamentous structures in all three isolates. According to Justice et al. (2008), bacterial cells exhibit filamentous morphology that enables them to survive under environmental stressors such as antimicrobial agents and host effectors. In their study, Bos et al. (2015) showed that exposure to sub-MIC ciprofloxacin leads to a stimulation of the SOS response and the formation of long filaments containing multichromosome in *E. coli* cells. They also found that new *E. coli* generations that are resistant to ciprofloxacin arise from asymmetric division of these multichromosomal filaments at their ends. They also reported that this new generation resistant to ciprofloxacin may have arisen from mutant chromosomes created by recombination between chromosomes in the long filament structure.

Sub-MICs can affect the expression of bacterial virulence genes and lead to various genetic processes such as conjugation and mutagenesis (Ding et al. 2022; Sanz‐García et al. 2022). In this study, we investigated the effects of sub-MICs on *rfaH* gene expression of isolates. *rfaH* is a transcriptional regulatory protein that affects operons (such as *rfb*, *rfa*, *tra*, *hly*, *kps*, *cps*, and *chu*) responsible for the expression of many virulence factors, including O-antigen, LPS core, F-factor, alpha-haemolysin, group -I, -II, -III capsules, and hemin receptor (Nagy et al. 2002). According to Liu et al. (2019), an increase in *rfaH* expression can induce conjugation in bacteria via the *tra* operon pathway or enable the survival of the cell by inducing *bla*_*CTX-M-1*_, which hydrolyses beta-lactams. In this study, we observed that sub-MICs of antibiotics led to a significant increase in the expression of the *rfaH* gene in *R. ornithinolytica* and *K. quasivariicola*. This increase in the expression of the *rfaH* gene may have led to an increase in the expression of many virulence genes. When bacteria start to conjugate more frequently, this can lead to a rapid spread of antibiotic-resistant bacteria. The increase in antibiotic resistance among opportunistic pathogens is a growing concern. Recent studies indicate that sub-MIC antibiotics may increase the expression of virulence genes in opportunistic pathogens, posing a threat to public health (Navidifar et al. 2019; Shi et al. 2019; Haldar et al. 2023).

## Conclusion

The issue of antibiotic-resistant bacteria is of great concern to the public. The exposure of bacteria to sub-MICs of antibiotics is one of the most important factors for the development of antibiotic-resistant bacteria. (Baquero and Coque 2014). Therefore, it is very important to understand the effects of sub-MICs of antibiotics on the virulence factors of bacteria. It is known that antibiotic resistance increases rapidly in Enterobacteriaceae. However, there is no study on the effects of sub-MICs on the opportunistic pathogens of this family, namely *R. ornithinolytica*, *P. agglomerans*, and *K. quasivariicola*. In this study, we demonstrated that sub-MICs of antibiotics decreased the growth rate of these bacterial species, decreased siderophore and biofilm production, increased the production of surface polysaccharides in *R. ornithinolytica* and *P. agglomerans*, induced significant changes in bacterial morphology, especially with meropenem and ciprofloxacin, and upregulated the expression of *rfaH*, a regulator of many virulence operons in bacteria. These changes in virulence properties can lead to bacteria becoming resistant to antibiotics and this resistance can be transmitted. This in vitro study, conducted specifically for these three Enterobacteriaceae species, requires in vivo studies for a more comprehensive understanding of the mechanisms that may lead to the development of antibiotic resistance in the isolates in interest under sub-MIC conditions.

## Data Availability

The datasets generated during and/or analysed during the current study are available from the corresponding author on reasonable request.
